# Intensity of end-of-life health care and mortality after systemic anti-cancer treatment in patients with advanced lung cancer

**DOI:** 10.1186/s12885-021-07992-5

**Published:** 2021-03-15

**Authors:** Kersti Oselin, Heti Pisarev, Keit Ilau, Raul-Allan Kiivet

**Affiliations:** 1 Department of Chemotherapy, Clinic of Haematology and Oncology, North Estonia Medical Centre, J. Sütiste tee 19, 13419 Tallinn, Estonia; 2 Institute of Family Medicine and Public Health, Tartu University, Tartu, Estonia; 3 Pharmacy, North Estonia Medical Centre, Tallinn, Estonia

**Keywords:** Advanced lung cancer, End-of-life care, High intensity care

## Abstract

**Background:**

We aimed to study the mortality and intensity of health care in patients with advanced lung cancer who received systemic anti-cancer treatment (SACT) compared with patients who were not eligible for SACT (no-SACT).

**Methods:**

A retrospective cohort of patients with lung cancer, who were treated at the North Estonia Medical Centre from 2015 to 2017, was linked to population-based health care data from the Estonian Health Insurance Fund. We calculated 14- and 30-day mortality after SACT and used a composite measure of intensity of care, comprised from the following: emergency department visit, admission to hospital, admission to intensive care unit, receipt of radiotherapy or systemic treatment.

**Results:**

The median overall survival (OS) of patients who received at least one cycle of SACT (*n* = 489) was 9.1 months and in patients with no-SACT (*n* = 289) 1.3 months (hazard ratio [HR] = 4.23, 95% CI = 3.60–5.00). During the final 30 days of life, intensive EOL care was received by 69.9% of the SACT patients and 43.7% of the no-SACT patients. Intensive EOL care in the last 30 days of life is more probable among patients in the SACT group (odds ratio [OR] = 3.58, 95% CI = 2.54–5.04, *p* <  0.001), especially in those with a stage IV disease (OR = 1.89, 95% CI = 1.31–2.71, *p* = 0.001). In the SACT group 6.7 and 14.7% of patients died within 14 days and 30 days after the last cycle, respectively.

**Conclusions:**

Significant proportion of patients with advanced lung cancer continue to receive intensive care near death. Our results reflect current patterns of EOL care for patients with lung cancer in Estonia. Availability of palliative care and hospice services must be increased to improve resource use and patient-oriented care.

**Supplementary Information:**

The online version contains supplementary material available at 10.1186/s12885-021-07992-5.

## Background

The intensity of treatment towards the end of life (EOL) has been suggested as one of the factors most affecting quality of life (QoL) in patients with advanced cancer [1, 2]. Intensive care at the EOL is variably defined in literature. In 2003, Earle and colleagues were first to identify several markers of potentially overly aggressive EOL cancer care, later several studies supported these conclusions [3–5]. The internationally recognised intensity of care and quality of EOL care indicators in oncology are: intensive use of systemic anti-cancer treatment (SACT), low rates of hospice use, ED visits, hospitalisations and admissions to ICU; all measures occurring within 14 or 30 days of death [6–8]. These service-based indicators could easily be applied to existing administrative data to assess the utilisation of health care services at the EOL retrospectively.

ASCO’s expert panel for the “Choosing Wisely” campaign identified the use of chemotherapy with unknown benefits and in patients with poor performance status as the most widespread unnecessary practice in oncology [9]. Studies have reported that 5–22% of patients with advanced stage cancer received SACT within 2 weeks of death and up to 55% in the last month of life [10, 11]. Early post-treatment mortality may be associated with SACT-related toxicity. Previous studies assessing chemotherapy use at the EOL, however, have not always been able to discriminate between treatment-related and cancer-related mortality.

Despite recent developments in immunotherapy, conventional chemotherapy remains the cornerstone of the treatment of advanced stage lung cancer. Neutropenia and infectious complications are common and potentially life-threatening adverse effects of chemotherapy. As shown in the recent study by Whitney et al., the most common diagnoses resulting in unplanned hospitalisations through ED visits included infection or fever, and among patients with different solid tumours the rates were highest for lung cancer, with 5.2% of all cancer-related hospitalisations being related to neutropenia or fever in the US [12, 13].

In Estonia, approximately 800 people annually are newly diagnosed with lung cancer [14]. In the current study, we aimed to characterise EOL care, health care intensity in the final 30 days of life and SACT-related mortality with an emphasis on infectious complications in patients with advanced stage lung cancer based on routine clinical data from the North Estonia Medical Centre’s Thoracic Oncology Database. The Estonian Cancer Registry has been collecting data on cancer incidence and mortality since 1968, but in regard to anti-cancer treatment only surgery is captured. Particularly, data on palliative care in Estonia are lacking. We provide data that will inform oncologists and policymakers of the limitations in lung cancer care and should help them plan interventions to improve the quality of care.

## Methods

### Study cohort

**Fig. 1:**
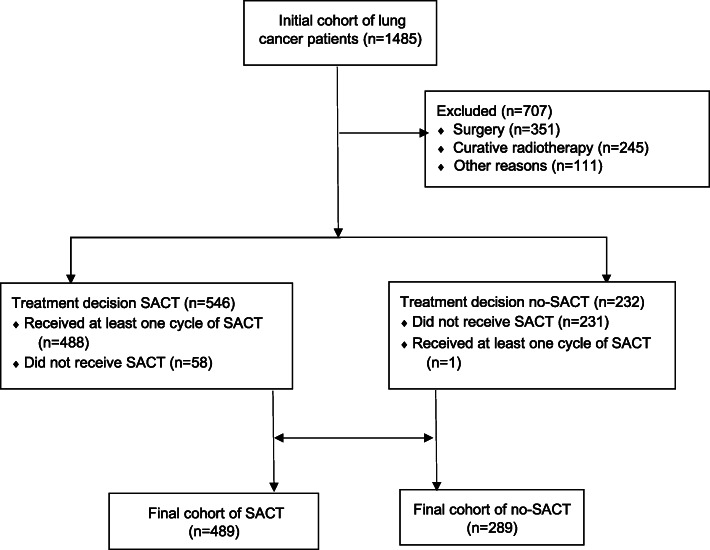
Flow chart of the study population. Abbreviations: SACT, systemic anti-cancer treatment

This is a retrospective analysis of the health care data of patients with lung cancer treated at the North Estonia Medical Centre between 1 January 2015 and 31 December 2017. The North Estonia Medical Centre is the single provider of all types of oncology services for a population of 800,000, and its Thoracic Oncology Database has covered all patients with lung cancer since 2015. During the study period, a multidisciplinary tumour board had confirmed treatment decisions of 1485 patients with lung cancer (Fig. 1). To identify the impact of palliative SACT, we excluded patients with lung cancer with local disease whose primary treatment was either surgery or radiotherapy, irrespective of whether this was combined with SACT; 111 patients excluded had either duplicate decisions or patient refusal of any investigations. This cohort contained 778 patients: 489 received SACT (SACT group) and 289 were not eligible to receive SACT (no-SACT group). The study was approved by the Tallinn Ethics Committee for Medical Research (no. 1972).

### Data sources

Patient characteristics such as age, sex, stage, date of biopsy if performed, histology, disease status as newly diagnosed or recurrent and date of treatment decision were extracted from the Thoracic Oncology Database. The patient’s national identification code from the Thoracic Oncology Database was linked to the electronic database of billing data of the Estonian Health Insurance Fund. This database incorporates detailed data on all medical services used during a hospital stay and any out-patient visits, including each cycle of SACT provided. To calculate the chemotherapy receipt within the last 14 and 30 days of life, the date of last cycle of SACT was linked to the date of death. The electronic data of patients who died within 14 and 30 days after the last cycle of SACT were reviewed in detail by two authors independently, and the probable cause of death related to progression and SACT-related toxicity such as sepsis, bacterial infection and neutropenia was identified. Data on the death, if applicable, was retrieved from the National Death Registry. The data cut-off date was 31 July 2018.

### Outcome measures

The outcome measures to be assessed in this study were chosen on the basis of previous research. We analysed health care utilisation and the circumstances of death, calculated 14- and 30-day mortality after SACT and used a composite measure of intensity of care, comprised from the following: emergency department visit, admission to hospital, admission to ICU, receipt of radiotherapy or systemic treatment (except no-SACT). For comorbidities in the whole cohort, we used only major diagnoses affecting chemotherapy administration such as diabetes (type 1 and 2), atrial fibrillation, cardiovascular disease (cardiac and peripheral vascular disease), and chronic obstructive pulmonary disease.

### Statistical analysis

To describe the baseline characteristics and background data of the study population and health care use at the EOL, frequencies and percentages were used for categorical data and mean values with standard deviations (SD) or median values with quartiles (Q25-Q75) for numeric data. To compare the characteristics of SACT and no-SACT patients and SACT patients who died within 14 days and 30 days after their last treatment, the Mann-Whitney U test (numeric variables), Fisher test or z-test (categorical variables) was used. Overall survival (OS) was calculated from the treatment decision date. We used Kaplan Meier estimates to evaluate the survival of patients with SACT versus those with no-SACT. A multivariate Cox proportional hazards model was used to evaluate the effect of chemotherapy, sex, age, stage, disease status, histology, and comorbidities on survival among patients with SACT. The same variables were studied as potential predictors of intensive EOL care; statistical significance was tested with the z-test. Multivariable logistic regression models were constructed to determine factors associated with intensive EOL care. All significance tests were two sided with an α-level of 0.05. Bonferroni corrections were used on multiple comparisons. Patients were censored at the time of the data cut-off date and assumed alive if no national death date was given. All analyses were conducted using Stata 14.2 software.

## Results

### Characteristics of the study population and treatment patterns

A retrospective cohort of patients with lung cancer, who were treated at the North Estonia Medical Centre during the study period from 2015 to 2017, was assembled using the institutional Thoracic Oncology Database (Fig. 1). Patients’ baseline characteristics are presented in Table 1. The patients in the SACT group were younger than those who did not receive SACT. A biopsy was performed in almost all patients of the SACT group, whereas it was not possible in 23% of no-SACT patients. As a result, no histology data was available for 47% of no-SACT patients vs 15% of SACT patients. The patients in the no-SACT group had more cardiac comorbidities, whereas the patients in the SACT group had more treatment-related complications. In the SACT group, the first cycle of SACT was administered on average 24 days after the treatment decision. The mean number of SACT courses was 6.3 (median 4), and the patients with adenocarcinoma received twice as many SACT courses compared with squamous and small-cell lung cancer patients (Supplemental Table 1).

**Table 1:** Baseline and clinical characteristics of the study population

	SACT (***n*** = 489)	no-SACT (***n*** = 289)	***p***
**Male**	338 (69.1%)	219 (75.8%)	0.049
**Female**	151 (30.9%)	70 (24.2%)	
**Age at the time of MDT**			< 0.001
≤ 49.9	20 (4.1%)	5 (1.7%)	
50–64.9	194 (39.7%)	45 (15.6%)	
65–74.9	183 (37.4%)	115 (39.8%)	
≥ 75	92 (18.8%)	124 (42.9%)	
median (Q25–Q75)	66 (60–73)	73 (67–79)	
**Biopsy**			
Yes	483 (98.8%)	222 (76.8%)	< 0.0001
No	6 (1.2%)	67 (23.2%)	
**Histology**			< 0.001
adenocarcinoma	168 (34.4%)	45 (15.6%)	
squamous	124 (25.4%)	69 (23.9%)	
small cell	125 (25.6%)	40 (13.8%)	
Other/no malignancy/no histology	72 (14.7%)	135 (46.7%)	
**Stage**			0.74
II	2 (0.4%)	0 (0%)	
III	137 (28.0%)	80 (27.7%)	
IV	350 (71.6%)	209 (72.3)	
**Newly diagnosed**	390 (79.8%)	252 (87.2%)	0.008
**Recurrence**	99 (20.2%)	37 (12.8%)	
**Time from biopsy to MDT (days)**			
median (Q25–Q75)	12 (2–27)	8 (0–18)	0.0005
(min–max)	(0–3555)	(0–3240)	
**Palliative radiotherapy**			
*≤ 3 months before MDT*	14 (2.9%)	6 (2.1%)	0.64
*since MDT*	181 (37.0%)	16 (5.5%)	< 0.001
**Comorbidities**			
*≤ 3 months before MDT*			
Sepsis	1 (0.2%)	2 (0.7%)	0.56
Bacterial infection	0 (0%)	4 (1.4%)	0.019
Drug-related neutropenia	0 (0%)	0 (0%)	
Diabetes	54 (11.0%)	42 (14.5%)	0.176
Atrial fibrillation	49 (10.0%)	53 (18.3%)	0.001
Cardiovascular disease	266 (54.4%)	187 (64.7%)	0.005
COPD	107 (21.9%)	85 (29.4%)	.02
*since MDT*			
Sepsis	52 (10.6%)	16 (5.5%)	0.018
Bacterial infection	44 (9.0%)	3 (1.0%)	< 0.001
Drug-related neutropenia	34 (7.0%)	0 (0%)	< 0.001
COPD	109 (22.3%)	13 (4.5%)	.001

Abbreviations: *MDT* Multidisciplinary tumour board; *SACT* Systemic anti-cancer treatment; *Q* Quantile; *COPD* Chronic obstructive pulmonary disease

### Overall survival and location of death

**Fig. 2:**
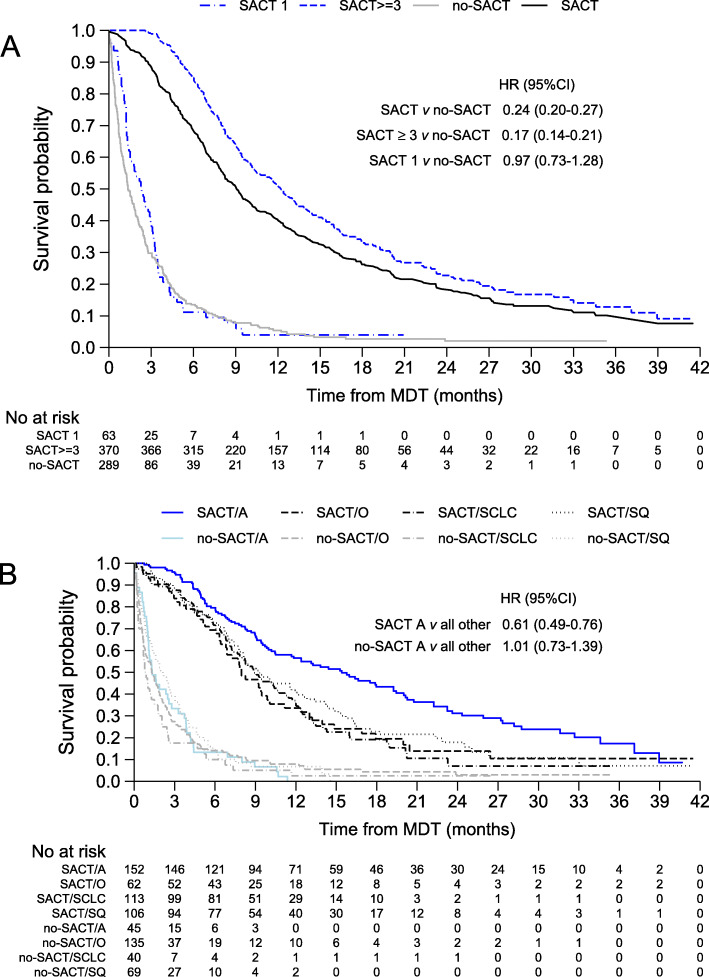
Overall survival in the study population (panel **a**) according to number of SACT cycles and (panel **b**) histology. Abbreviations: SACT, systemic anti-cancer treatment; MDT, multidisciplinary tumour board; A, adenocarcinoma; O, all other histologies combined; SCLC, small cell lung cancer; SQ, squamous cell; HR, hazard ratio

By the study cut-off date, 77% of SACT patients had died compared with 96% of no-SACT patients (Table 2). The median OS of no-SACT patients was 1.3 months (Q25-Q75 0.5–3.5), in 58 patients who received no SACT despite the MDT decision median OS was 1.6 months (Q25-Q75 0.7–4.6). In patients who received at least one cycle of SACT OS was 9.1 months (hazard ratio [HR] = 4.23, 95% CI = 3.60–5.00, *p* <  0.001), (Fig. 2**,** Panel a). The OS of patients with adenocarcinoma histology was significantly longer than all other histologies (median OS 13.3 vs 8.1 months, HR = 0.61, 95% CI = 0.49–0.76, *p* <  0.001), but there was no difference in the OS between histological subtypes in the no-SACT group (*p* = 0.951), (Fig. 2**,** Panel b). Among the patients who died during the study period, only 21% of patients in the no-SACT group died in a hospital (acute care or nursing hospital), whereas 38% of the SACT group deaths occurred in an acute care hospital and 24% in a nursing hospital (Table 2). In total, 22% of patients in the SACT group died in the hospital after they had been admitted via emergency department. In the SACT group, 77% of the deaths had occurred at the data cut-off date with a median survival of 75 days after the last cycle of SACT (Supplemental Table 1).

**Table 2:** Mortality and location of death in patients with and without systemic anti-cancer treatment

	SACT (***N*** = 489)	No-SACT (***N*** = 289)
Died ≤14 days since MDT, N (%)	4 (0.8)	68 (23.5)
Died ≤30 days since MDT, N (%)	12 (2.5)	118 (40.8)
Died ≤30 days since last cycle, N (%)	72 (14.7)	NA
Died at data cut off, N (%)	376 (76.9)	279 (96.5)
Place of death		
Acute care hospital^a^	144 (38.3)	41 (14.2)
*Following ED attendance* ^b^	82 (21.8)	*28* (*9.7*)
Nursing home	90 (23.9)	20 (6.9)
Out-of-hospital	142 (37.8)	218 (75.4)

Abbreviations: *SACT* Systemic anti-cancer treatment, *ED* Emergency department

^a^Died at hospital when the date of last claim was the date of death plus 1 day

^b^Died at hospital through ED visit when the last claim started with the date of ED claim. 9 patients in the no-SACT arm and 30 patients in the SACT arm died on same or next day as ED visit

### Intensity of care at the EOL

Any one of the following outcomes – hospitalisation, ED visit, ICU admission, radiotherapy or SACT (except no-SACT) – occurred during the final 30 days of life in 70% of the SACT group and 44% of the no-SACT group (Table 3). 0.8 and 2.5% of SACT patients had died within 14 and 30 days after the treatment decision, respectively, compared with the 24 and 41% in the no-SACT group (Table 2). Only 22 (7.6%) of patients in the no-SACT group received palliative RT, of whom seven (31.8%) died within 30 days (Table 2). In total, 195 (39.9%) patients in the SACT group received radiotherapy, but only 12 patients (6.2%) received radiotherapy within the final 30 days of life. The 63 patients who received only one course of SACT had a similar OS to patients with no-SACT (HR = 0.97, 95% CI = 0.73–1.28, *p* = 0.8), (Fig. 2**,** Panel a), indicating no benefit from systemic treatment in these patients. An additional 37 patients died within 30 days after the last cycle of SACT due to disease progression (Table 4). Combined, this results in a total of 100 patients for whom the final cycle of SACT undergone did not provide benefit and may be considered overtreatment. Intensive EOL care in the last 30 days of life is more probable among patients in the SACT group (odds ratio [OR] = 3.58, 95% CI = 2.54–5.04, *p* <  0.001), especially in those with a stage IV disease (OR = 1.89, 95% CI = 1.31–2.71, *p* = 0.001).

**Table 3:** The intensity of care in the final 30 days of life in deceased patients

Care measure	SACT (***N*** = 376)		No-SACT (***N*** = 279)		***p***^c^
	N	%	N	%	
Any hospitalization	237	63.0	110	39.4	< 0.001
Hospitalizations^a^	186	49.5	99	35.5	< 0.001
*Sepsis*	42	11.2	11	3.9	0.0064
*Bacterial infection*	14	3.7	1	0.4	0.0352
*Neutropenia*	11	2.9	0	0.0	–
ED visit	187	49.7	87	31.2	< 0.001
ICU admission	49	13.0	36	12.9	0.99
SACT	72	19.1	–	–	–
RT	12	3.2	7	2.5	0.99
Composite of measures^b^	263	69.9	122	43.7	< 0.001
All 5 (SC 4)	0	0.0	0	0.0	
None	113	30.1	157	56.3	< 0.001

Abbreviations: *SACT* Systemic anti-cancer treatment, *ED* Emergency department, *ICU* Intensive care unit, *RT* Radiotherapy

^a^Hospitalizations without sepsis, bacterial infection or neutropenia

^b^Any of the following outcomes: hospitalization, ED visit, ICU admission, SACT (except SC), or RT

^c^Tested by z-test with Bonferroni correction

**Table 4:** Cause of death in patients who died within 14 days or during 15–30 days of systemic anti-cancer treatment

Characteristics	≤ 14 days ***N*** = 33	15–30 days ***N*** = 39	Total ***N*** = 72	***p***
Died after 1st SACT cycle, N (%)	16 (48.5)	9 (23.1)	25 (34.7)	**0.03**
Total SACT cycles (median, range)	2 (1–13)	4 (1–17)	3 (1–17)	0.06
NEU before last cycle, N (%)	5 (15.2)	1 (2.6)	6 (8.3)	0.09
GCS-GF^a^	3 (9)	3 (7.7)	6 (8.3)	1
NEU/PAN after last cycle	9 (27.3)	5 (12.8)	14 (19.4)	0.145
ICU before death	15 (45.5)	5 (12.8)	20 (27.8)	**0.003**
Cause of death, N (%)				0.09
CV	3 (9.1)	3 (7.7)	6 (8.3)	
INF	9 (27.3)	7 (18)	16 (22.2)	
PD	13 (39.4)	26 (66.7)	39 (54.2)	
SUDDEN	8 (24.2)	3 (7.7)	11 (15.3)	

Abbreviations: *SACT* Systemic anti-cancer treatment, *NEU* Neutropenia, *INF* Infection, *PD* Progression, *GCS-GF* Granulocyte colony stimulating growth factor, *PAN* Pancytopenia, *ICU* Intensive care unit admission, *CV* Cardiovascular, *SUDDEN* sudden death or unknown with good performance status at the time of last SACT

^a^GCS-GF primary prophylaxis before last cycle. Values in bold are significant at *p* < 0.05

### SACT-related toxicity and 14- and 30-day mortality

The SACT patients received on average 6.3 (median 4) cycles of systemic chemotherapy and the median duration of treatment was 78 days, except for patients with adenocarcinoma (on average 9 courses over 135 days; *p* <  0.001), (Supplemental Table 1). In the SACT group, 10.6 and 7.0% of patients developed at least one case of sepsis or neutropenia, respectively (Table 1). In the SACT group, 33 patients (6.7%) died within 14 days and 72 patients (14.7%) within 30 days of the last SACT cycle. 11.2% of the deceased were hospitalised due to sepsis and 2.9% had neutropenia in the final 30 days (Table 3). In total, 46% of early deaths (in 14 days) occurred after the first cycle of SACT. 22.2, 6.9 and 11.1% of patients with 30-day mortality after SACT developed sepsis, bacterial infection or drug-related neutropenia, respectively, but were less frequent in patients who died more than 30 days after the last cycle of SACT (Supplemental Table 2). In the multivariate Cox proportional hazard models, male sex, age younger than 69 years, histology other than adenocarcinoma had a significant adverse impact on survival (Table 5). We then aimed to identify the cause of death in patients with early SACT-related mortality. Patients who died within 14 days after SACT were more likely to die after the first cycle (*p* = 0.03), to have one episode of neutropenia before the last cycle (15.2% vs 2.6%, *p* = 0.09), to develop neutropenia or pancytopenia after the last cycle (27.3% vs 12.8%, *p* = 0.145), to be admitted to the ICU before death (*p* = 0.003) and to die due to infection (*p* = 0.09) (Table 4).

**Table 5:** Multivariate Cox proportional hazards models of survival among patients who received systemic anti-cancer treatment

Characteristic	Unadjusted HR (95%CI)	Adjusted^a^ HR (95%CI)	Adjusted ***p***
**Sex**			
Female	1	1	
Male	**1.56 (1.24–1.96)**	1.38 (1.08–1.76)	**0.011**
**Age**			
≤ 49	1	1	
50–59	**2.11 (1.14–3.89)**	2.2 (1.18–4.08)	**0.013**
60–69	**1.80 (1.00–3.25)**	1.87 (1.02–3.4)	**0.042**
70–79	1.45 (0.80–2.64)	1.58 (0.86–2.92)	0.14
80 ≥	1.67 (0.84–3.31)	1.93 (0.96–3.88)	0.065
**Comorbidities (yes vs no)**			
Diabetes	0.94 (0.71–1.26)	1.06 (0.78–1.43	.716
Cardiovascular	0.84 (1.05–1.43)	0.89 (0.69–1.14)	.347
Atrial fibrillation	1.00 (0.78–1.30)	0.99 (0.75–1.31)	.956
COPD	0.99 (0.80–1.25)	0.9 (0.71–1.13)	0.353
**Disease status**			
Newly diagnosed	1.14 (0.88–1.47)	1.26 (0.96–1.65)	**.009**
Recurrence	1	1	
**Stage**			
II	0.87 (0.21–3.52)	1.18 (0.28–4.98)	.824
III	1	1	
IV	0.96 (0.76–1.20)	1.11 (0.87–1.4)	.402
**Histology**			
Adenocarcinoma	1	1	
Squamous	**1.58 (1.21–2.06)**	**1.54 (1.15–2.08)**	**.004**
SCLC	**1.63 (1.19–2.24)**	**1.48 (1.07–2.05)**	**.019**
Other	**1.70 (1.29–2.24)**	**1.59 (1.18–2.14)**	**.002**
**Other cancer treatment**			
Radiotherapy	1.02 (0.83–1.26)	0.9 (0.72–1.12)	.334

*HR* Hazard ratio, *CI* Confidence interval, *p* Probability, *COPD* Chronic obstructive pulmonary disease, *SCLC* Small cell lung cancer

^a^Adjusted on all patient and disease related variables in the table. Values in bold are significant at *P* < .05

## Discussion

To our knowledge, this is the first study to evaluate EOL cancer care in Estonia and in Eastern Europe. Our results confirm that a significant proportion of patients with advanced lung cancer continue to receive intensive care near death. Nearly 15% of patients in the current study died within 30 days after the last cycle of SACT. Infectious complications and neutropenia were responsible for over one in five of these deaths. These findings are particularly important, as chemotherapy in combination with immunotherapy is the currently recommended first line of treatment in most patients with advanced lung cancer.

Patients with a poor performance status do not benefit from chemotherapy [9, 15]. In our study, patients with the treatment decision no-SACT had a poor prognosis with a median OS of 1.3 months. Due to the lack of EOL services, they were frequently hospitalised through ED visits near death. 78% of the deceased in the no-SACT group died outside hospital (acute or nursing hospital). There was no possibility to collect data on care and costs paid by a family, including home-based care. However, our findings indicate very limited provision of organised health care services to patients deemed ineligible for active oncological treatment. It is well recognised that EOL care is a subject for considerable policy differences, which also exist among developed countries. For instance, in the US and the Netherlands, the lowest proportion of cancer patients died in acute care hospitals (less than 30%), whereas 77% of cancer patients in France die in hospitals [16, 17]. Previous studies have not reported EOL data depending on patients’ eligibility for active oncological treatment; however, this likely affects who provides EOL care and how it is provided. Our study demonstrated that there is an urgent need to increase EOL supportive care services in Estonia, particularly for patients not eligible for active treatment.

The median OS of patients in this study who received at least one cycle of SACT was 9.1 months. One-third of patients who opted for SACT accounted for potential overtreatment, including 63 (13%) patients who received only one cycle of SACT, 37 (7%) patients who died of progression after receiving SACT in the final 30 days and 58 (11%) patients who never received systemic treatment despite the decision. Timely access to SACT is an important aspect of cancer care, particularly affecting patients with high disease and symptom burden. The median time from the diagnosis to the start of systemic treatment was approximately 1 month in the current study. Prioritisation of patients eligible for SACT is necessary to reduce delays in treatment initiation and improve outcome. Of those who did not undergo SACT OS was 1.3 months. 22 (7.6%) of these patients received palliative radiotherapy with seven (31.8%) dying within 30 days. In light of their very short survival and the anticipated time to treatment benefit, it is unlikely that these patients will have benefitted from radiotherapy.

We chose previously reported indicators of the intensity of cancer care relevant to adult patients with solid tumours [3–8]. A composite measure for intensive EOL care was received by 70% of the deceased SACT patients and 44% of the no-SACT patients in the current study. Moreover, 66% of the total 489 SACT patients had at least one ED visit after the initiation of SACT, including 30 patients who died on the same or next day as the ED visit. Our findings are in line with those from Canada, where 62% of patients with lung cancer had at least one ED visit in the last 30 days [17]. In the US, ICU admissions near death were twice as high as those in six other developed countries, with 27% of adult cancer patients being admitted to the ICU in the last 30 days [17]. In our study, 13% of patients in both the SACT and no-SACT groups experienced ICU admission. We found no evident pattern between patient characteristics and the likelihood of receiving intensive EOL care. Future research should focus on causes leading to ED visits, whether these could be avoided by improving access to oncology services directly 24/7 and palliative and hospice care.

Of the various intensive EOL care measures, the use of chemotherapy near death has been studied most extensively. Chemotherapy use in the last 30 days of life ranged from 4.8% (Norway) to 10.6% (US) and 12.7% (Belgium) [17]. Among various solid tumours, patients with lung cancer were more likely to receive chemotherapy at the EOL [18]. At the data cut-off date, 19% of the deceased patients in our study had received SACT in the final 30 days of life, similar to the rate reported in France [16]. In England, 30-day mortality after SACT was higher in patients with lung cancer compared to patients with breast cancer, with a considerable difference in curative (3%) and palliative (10%) treatments [19]. Infectious complications were significant predictors for early SACT-related death and hospitalisations in our study (Supplemental Table 2). Among EOL hospitalisations, 11% were due to sepsis and 3% of patients had neutropenia. Patients who died within 14 days after SACT were more likely to die due to neutropenia and infection, whereas patients who died within 15–30 days after the last cycle of SACT were more likely to die of progressive disease. It has previously been shown that cancer-related neutropenia accounts for a substantial amount of the total cancer-related hospitalisation costs [20]. Using the same dataset for our ongoing study, we found that neutropenia and severe infection resulted in median hospital stay of 11 days in our hospital. ASCO’s guideline for the management of neutropenia generally recommends primary prophylaxis when the risk of neutropenia is high (> 20%) [21]. In recent decade, several biosimilar GCS-GF products have been approved in the EU and in rest of the world, making the use of neutropenia prophylaxis more cost-effective. Our findings raise the possibility that a lower threshold for the prophylactic use of GCS-GF may be needed for lung cancer.

Our study has several limitations. Firstly, it was a single centre retrospective cohort study with a relatively small sample size. Secondly, our study was based on the data of health service use and we had no information on the performance status of patients. However, for those patients who died within 30 days after SACT, a detailed review of electronic medical records was conducted and the probable cause of death identified. The strengths of this study include: a) universal insurance coverage in Estonia, meaning our results capture all health care services provided to patients; b) systemic treatment of lung cancer for the whole population in the catchment area is delivered only in this specific cancer centre, thus the study cohort includes all cancer patients; c) homogenous population, eg patients with advanced lung cancer from the routine clinical database; d) homogenous systemic treatment, as ALK-inhibitors and immunotherapy were not reimbursed at the time of the study; e) most recent study period; f) the cause of death was evaluated through detailed review of electronic medical records.

## Conclusions

A significant proportion of patients with advanced lung cancer continue to receive intensive care near death with a negative health impact. Progressive disease was the main cause of death. Chemotherapy is the cornerstone of treatment for advanced lung cancer and our results illustrate that careful selection of patients for SACT is important. Deaths due to neutropenia and infectious complications constituted a clinically significant proportion of those dying within 30 days. Combined with the ED attendances shown here this finding supports the need for improved support for patients on SACT. Finally, despite evidence of careful selection for SACT in many cases, our results reflect the patterns of the EOL care of lung cancer in Estonia, where the availability of palliative health care services and hospice care must be increased to improve resource use and patient-oriented care.

## Supplementary Information

**Additional file 1: Supplemental Table 1**. Use of systemic anti-cancer treatment in patients with advanced lung cancer. **Supplemental Table 2**. Myelotoxicity and infectious complications in patients with and without 14- and 30-day mortality after last cycle of systemic anti-cancer treatment.

## Data Availability

Datasets analyzed are available from the corresponding author.

## Abbreviations

ED: Emergency department
EOL: End of life
ICU: Intensive care unit
GCS-GF: Granulocyte colony stimulating growth factor
MDT: Multidisciplinary tumour board
OS: Overall survival
QoL: Quality of life
SACT: Systemic anti-cancer treatment
