# Supplementary material for: Intensity of end-of-life health care and mortality after systemic anti-cancer treatment in patients with advanced lung cancer
Source: BMC Cancer. 2021 Mar 15;21:274. doi: 10.1186/s12885-021-07992-5 (PMC7958422; doi:10.1186/s12885-021-07992-5)
Supplement: Supplementary file 1 — Additional file 1: Supplemental Table 1. Use of systemic anti-cancer treatment in patients with advanced lung cancer. Supplemental Table 2. Myelotoxicity and infectious complications in patients with and without 14- and 30-day mortality after last cycle of systemic anti-cancer treatment. [file 12885_2021_7992_MOESM1_ESM.docx]

**Supplemental data**

**Table 1. Use of systemic anti-cancer treatment in advanced lung cancer patients.**

|  | **All**  **(N=489)** | **Adenocarcinoma**  **(N=168)** | **Squamous**  **(N=124)** | **SCLC**  **(N=125)** | **Other**  **(N=72)** |
| --- | --- | --- | --- | --- | --- |
| **Number of cycles**  *mean/median/Q25/Q75* | 6.3/4/1/13 | 9.3/6/3/12  *Adeno v all other histologies*  **p < 0.001** | 5.1/4/2/6  *SQ v SCLC/Other*  **p > 0.05** | 4.4/4/3/6 | 4.5/4/2/5 |
| **Patients with N (%)**  **1 cycle**  **2 cycles**  **≥3 cycles** | 63 (12.9%)  56 (11.5%)  370 (75.8%) | 13 (7.7%)  16 (9.5%)  139 (82.7%) | 19 (15.3%)  8 (14.5%)  87 (70.2%) | 17 (13.6%)  12 (9.6%)  96 (76.8%) | 14 (19.4%)  10 (13.9%)  48 (66.7%) |
| **Treatment lines**  **(≥** 90 days between cycles)  1  2  3  4 | 400 (81.8%)  80 (16.4%)  8 (1.6%)  1 (0.2%) | 132 (78.6%)  32 (19.1%)  3 (1.8%)  1 (0.6%) | 102 (82.3%)  19 (15.3%)  3 (2.4%)  0 (0%) | 103 (82.4%)  20 (16.0%)  2 (1.6%)  0 (0%) | 63 (87.5%)  9 (12.5%)  0 (0%)  0 (0%) |
| **Time from MDT to 1st cycle (days)**  *mean (SD)*  *median (Q25–Q75)*  *(min–max)* | 23.7 (17.7)  22 (16*–*28)  (0*–*84) | 24.2 (13.8)  23 (17*–29*)  (0*–*84) | 27 (27)  22 (17*–29*)  (0*–*268) | 21 (12)  20 (13*–27*)  (0*–*86) | 23(10)  23 (18*–27*)  (1*–*56) |
| **Duration of treatment (days)**  *mean (SD)*  *median (Q25–Q75)*  *(min–max)* | 162 (200)  78 (42–219)  (0*–*1162) | 251 (264)  135 (56*–407*)  (0*–*1162)  *Adeno v all other histologies*  **p < 0.001** | 122 (143)  69 (28*–151*)  (0*–*656)  *SQ v SCLC/Other* **p > 0.05** | 118 (130)  77 (46*–141*)  (0*–*849) | 101 (128)  68 (21*–124*)  (0*–*678) |
| **Time from last cycle till death**  *mean (SD)*  *median (Q25–Q75)*  *(min–max)* | 115 (128)  75 (38*–141*)  (0*–*917) | 119 (149)  70.5 (38*–131*)  (1*–*807) | 121 (132)  91 (38*–148*)  (9*–*917) | 104 (94)  75 (36*–142*)  (0*–*492) | 114 (124)  72 (44*–152*)  (0*–*779) |
| **Death (cut off 31.07.2018)**  **N (%)**  ≤ 14 days after last cycle  ≤ 30 days after last cycle | 376 (76.9)  33 (6.7)  72 (14.7) | 118 (70.2)  8 (4.8)  20 (11.9) | 103 (83.1)  6 (4.8)  21 (16.9) | 96 (76.8)  12 (9.6)  19 (15.2) | 59 (81.9)  7 (9.7)  12 (16.7) |

Abbreviations: SD, standard deviation; Q, quantile; p – probability; MDT, multidisciplinary tumour board; Adeno, adenocarcinoma; SQ, squamous; SCLC, small cell lung cancer. One patient with adenocarcinoma histology had initially refused treatment, the time from MDT to 1st cycle was 858 days, the patients was excluded from the calculations of mean, SD, and median. Significant p values (< 0.05) are shown in bold type.

**Table 2. Myelotoxicity and infectious complications in patients with and without 14- and 30-day mortality after last cycle of systemic anti-cancer treatment.**

|  | **Death ≤ 14 days** | |  | **Death ≤ 30 days** | |  |
| --- | --- | --- | --- | --- | --- | --- |
|  | **Yes**  **(n=33)** | **No**  **(n=456)** | **p** | **Yes**  **(n=72)** | **No**  **(n=417)** | **p** |
| **Age, N (%)**  ≤ 49.9  50–64.9  65–74.9  ≥ 75  median (Q25-Q75) | 0 (0)  13 (39.4)  14 (42.4)  6 (18.2)  66 (57–77) | 20 (4.4)  181 (39.7)  169 (37.1)  86 (18.9)  66 (60–73) | 0.78 | 1 (1.4)  32 (44.4)  27 (37.5)  12 (16.7)  65 (57–70) | 19 (4.6)  162 (38.8)  156 (37.4)  80 (19.2)  66 (60–73) | 0.61 |
| **Male, N (%)**  **Female, N (%)** | 22 (66.7)  11 (33.3) | 316 (69.3)  140 (30.7) | 0.84 | 53 (73.6)  19 (26.4) | 285 (66.4)  132 (31.6) | 0.41 |
| **Histology, N (%)**  Adenocarcinoma  Squamous  Small cell  Other | 8 (24.2)  6 (18.2)  12 (36.4)  7 (21.2) | 160 (35.1)  118 (25.9)  113 (24.8)  65 (14.2) | 0.22 | 20 (27.8)  21 (29.2)  19 (26.4)  12 (16.7) | 148 (35.5)  103 (24.7)  106 (25.4)  60 (14.4) | 0.59 |
| **Use of GCS-GF, N (%)**  *since MDT* | 9 (27.3) | 136 (29.8) | 0.198 | 22 (30.6) | 123 (29.5) | 0.89 |
| **SACT-related toxicity, N (%)**  *since MDT till death/cut off*  Sepsis  Bacterial infection  Drug-related neutropenia  *since last cycle till death/cut off*  Sepsis  Bacterial infection  Drug-related neutropenia | 6 (18.2)  3 (9.1)  5 (15.2)  3 (9.1)  1 (3.0)  5 (15.2) | 46 (10.1)  41 (9)  29 (6.4)  28 (6.1)  21 (4.6)  11 (2.4) | 0.146  1  0.069  0.486  1  **0.004** | 16 (22.2)  5 (6.9)  8 (11.1)  9 (12.5)  1 (1.4)  6 (8.3) | 36 (8.6)  39 (9.4)  26 (6.2)  22 (5.3)  21 (5.0)  10 (2.4) | **< 0.001**  0.18  0.31  **< 0.001**  0.99  0.18 |

*Duration of lung cancer was calculated from the date of biopsy if available for newly diagnosed patients, or from the date of MDT if no biopsy, and from the date of MDT for all patients with recurrence. MDT, multidisciplinary tumour board; GCS-GF –granulocyte colony stimulating growth factor; Q, quartile; SACT, systemic anti-cancer treatment. Significant p values (< 0.05) are shown in bold type.
